# Supplementary material for: The Importance of Interprofessional Collaboration (IPC) Guidelines in Stunting Management in Indonesia: A Systematic Review
Source: Healthcare (Basel). 2024 Nov 7;12(22):2226. doi: 10.3390/healthcare12222226 (PMC11593802; doi:10.3390/healthcare12222226)
Supplement: Supplementary file 1 [file healthcare-12-02226-s001.zip › healthcare-3231656-supplementary.pdf]

Review protocol: The Importance of Interprofessional Collaboration (IPC) Guidelines in Stunting Management in Indonesia: A Systematic Review

Background

Stunting remains a significant public health concern in Indonesia, affecting children under five years of age. Despite a decline in recent years, the prevalence of stunting was reported at 21.6% in 2022, which still constitutes a public health problem according to World Health Organization standards. Various stunting prevention programs have been implemented in Indonesia; however, their effectiveness remains suboptimal and lacks adequate scalability. Interprofessional collaboration (IPC) has been identified as crucial for preventing and treating pediatric stunting, emphasizing the need for a comprehensive approach involving healthcare professionals, community workers, and policymakers.

Objective

This systematic review aims to investigate the roles of primary health centers and interprofessional collaboration in addressing stunting in Indonesia. By synthesizing existing evidence, this review will provide valuable insights into the current state of stunting management, identify effective strategies, and highlight areas for improvement. The information collected will be crucial for policymakers, healthcare professionals, and researchers working to reduce stunting prevalence in Indonesia and potentially in other similar settings.

Review question

Full review question	
**What is the role and impact of interprofessional collaboration in stunting management within primary health centers in Indonesia, and how does it influence stunting prevention and treatment outcomes?**	
**Population**	Children under five years old in Indonesia, healthcare professionals, community health workers

**Intervention**	Interprofessional collaboration in stunting management
**Comparison**	Standard care or lack of interprofessional collaboration
**Outcome**	Stunting prevalence, effectiveness of prevention programs, healthcare utilization, and knowledge and skills of healthcare workers

Search strategy

Databases
• **PubMed**<br>• **EMBASE**<br>• **Web of Science**<br>• **ProQuest**<br>• **Google Scholar**
**Search terms**
Stunting OR stunted OR "growth disorder" OR malnutrition OR retardation OR "failure to thrive" OR "growth restriction" OR "growth retardation" OR "growth impairment"<br>AND<br>"interprofessional collaboration" OR "multidisciplinary" OR "interdisciplinary" OR "healthcare team" OR "primary health center" OR "community health"<br>AND<br>Indonesia
**Identifying other useful sources**
**Hand searching of key journals in nutrition, public health, and pediatrics**<br>**Checking reference lists of included studies**<br>**Searching websites of relevant organizations (e.g., WHO, UNICEF, Indonesian Ministry of Health)**<br>**Contacting experts in the field for unpublished or ongoing studies**

Eligibility criteria

PICO	Inclusion Criteria	Exclusion Criteria
Population	• Children under five years in Indonesia<br>• Healthcare professionals and community health workers involved in stunting management	• Children over five years<br>• Studies not conducted in Indonesia

Intervention	• Interprofessional collaboration in stunting management<br>• Primary health center-based interventions	• Single-profession interventions<br>• Hospital-based interventions
Comparison	• Standard care<br>• No interprofessional collaboration<br>• Before and after implementation of IPC	• Studies without a clear comparison group
Outcomes	• Stunting prevalence<br>• Effectiveness of prevention programs<br>• Healthcare utilization<br>• Knowledge and skills of healthcare workers	• Studies not reporting on at least one of the specified outcomes

Additional limits

Language: English and Indonesian

Publication type: peer-reviewed journal articles, government reports, and theses

Study design: randomized controlled trials, quasi-experimental studies, cohort studies, case-control studies, and cross-sectional studies

Publication date: no date restrictions

Study quality assessment

The Joanna Briggs Institute's critical appraisal tools will be used to assess the methodological quality of the included studies. Two independent reviewers will perform the quality assessment. Any disagreements will be resolved through discussion or consultation with a third reviewer.

Data extraction and synthesis

The data we extracted consisted of general study characteristics including (1) first author, (2) year of publication, (3) study design, and (4) title. We used the Joanna Briggs Institute's critical appraisal checklist to assess the risk of bias in the selected study. Two independent reviewers conducted individual assessments of each included study, and any disagreements were resolved through collaborative team discussions to reach a consensus.

Narrative techniques were employed for the analysis and synthesis of the data, a method highly advocated for the comprehensive analysis and synthesis of diverse data types in systematic reviews with mixed methodologies. The primary reviewer analyzed the extracted data, creating a textual narrative synthesis, which underwent further review and refinement in team discussions.
